# Ethyl 3-amino-1-phenyl-1*H*-benzo[*f*]chromene-2-carboxyl­ate

**DOI:** 10.1107/S1600536809008885

**Published:** 2009-03-14

**Authors:** Yuan-Hong Jiao, Qian Zhang, Fa-Yan Meng, Lei Teng, Jia Yuan, Seik Weng Ng

**Affiliations:** a School of Chemistry and Material Engineering, Huangshi Institute of Technology, Huangshi 435003, People’s Republic of China; b Department of Chemistry, University of Malaya, 50603 Kuala Lumpur, Malaysia

## Abstract

The pyranyl ring of the title compound, C_22_H_19_NO_3_, adopts a flattened-boat conformation. The dihedral angle between naphthalene and phenyl rings is 78.3 (1)°The mol­ecule also features an intra­molecular N—H⋯O_carbon­yl_ hydrogen bond. Adjacent mol­ecules are linked by an inter­molecular N—H⋯O_carbon­yl_ hydrogen bond, forming a zigzag chain that runs along the *c* axis.

## Related literature

For the crystal structures of other ethyl 3-amino-1-aryl-1*H*-benzo[*f*]chromene-2-carboxyl­ate derivatives, see: Klokol *et al.* (1987 ▶); Shi *et al.* (2003*a* ▶,*b* ▶); Wang *et al.* (2003 ▶); Zhuang *et al.* (2003*a* ▶,*b* ▶).
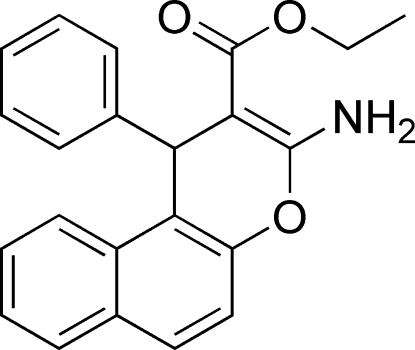


## Experimental

### 

#### Crystal data

C_22_H_19_NO_3_
*M* *_r_* = 345.38
Monoclinic, 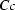

*a* = 13.7835 (5) Å
*b* = 14.6460 (4) Å
*c* = 8.8713 (2) Å
β = 99.551 (1)°
*V* = 1766.05 (9) Å^3^
*Z* = 4
Mo *K*α radiation
μ = 0.09 mm^−1^
*T* = 153 K
0.36 × 0.25 × 0.14 mm

#### Data collection

Rigaku R-AXIS RAPID diffractometer
Absorption correction: multi-scan (*ABSCOR*; Higashi, 1995 ▶) *T*_min_ = 0.864, *T*_max_ = 0.988
8563 measured reflections
2029 independent reflections
1958 reflections with *I* > 2σ(*I*)
*R*_int_ = 0.016

#### Refinement

*R*[*F* ^2^ > 2σ(*F* ^2^)] = 0.030
*wR*(*F* ^2^) = 0.078
*S* = 1.09
2029 reflections
244 parameters
4 restraints
H atoms treated by a mixture of independent and constrained refinement
Δρ_max_ = 0.19 e Å^−3^
Δρ_min_ = −0.27 e Å^−3^

### 

Data collection: *RAPID-AUTO* (Rigaku, 1998 ▶); cell refinement: *RAPID-AUTO*; data reduction: *CrystalStructure* (Rigaku/MSC, 2002 ▶); program(s) used to solve structure: *SHELXS97* (Sheldrick 2008 ▶); program(s) used to refine structure: *SHELXL97* (Sheldrick, 2008 ▶); molecular graphics: *X-SEED* (Barbour, 2001 ▶); software used to prepare material for publication: *publCIF* (Westrip, 2009 ▶).

## Supplementary Material

Crystal structure: contains datablocks global, I. DOI: 10.1107/S1600536809008885/zl2186sup1.cif

Structure factors: contains datablocks I. DOI: 10.1107/S1600536809008885/zl2186Isup2.hkl

Additional supplementary materials:  crystallographic information; 3D view; checkCIF report

## Figures and Tables

**Table 1:** Hydrogen-bond geometry (Å, °)

*D*—H⋯*A*	*D*—H	H⋯*A*	*D*⋯*A*	*D*—H⋯*A*
N1—H11⋯O3^i^	0.88 (1)	2.05 (1)	2.907 (2)	166 (2)
N1—H12⋯O3	0.88 (1)	2.07 (2)	2.707 (2)	129 (2)

Symmetry code: (i) 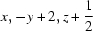.

**Fig. 1.**
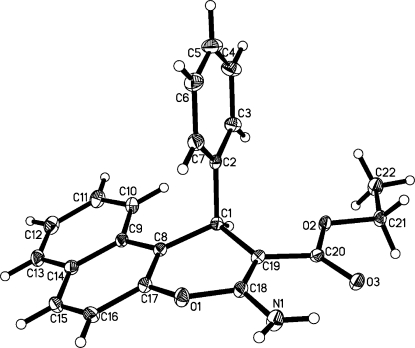
Thermal ellipsoid plot (Barbour, 2001) of C22H19NO3; displacement ellipsoids are drawn at the 70% probability level, and H atoms as spheres of arbitrary radius.

**Fig. 2.**
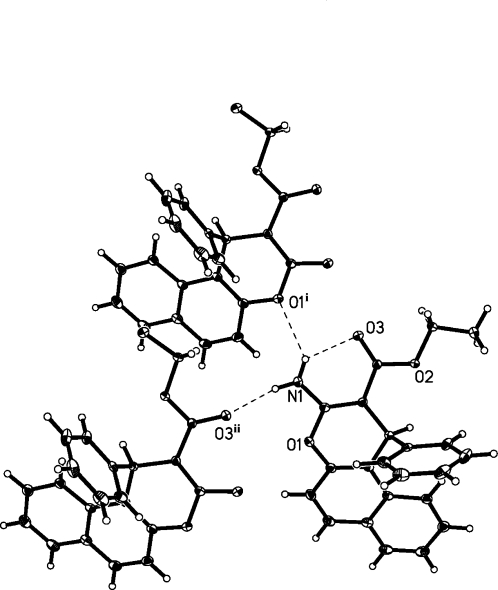
Thermal ellipsoid plot (Barbour, 2001) showing the intra and intermolecular hydrogen bonding interactions (dashed lines); displacement ellipsoids are drawn at the 70% probability level, and H atoms as spheres of arbitrary radius.

## supplementary crystallographic information

### Comment

2-Naphthol, substituted benzaldehydes and ethyl 2-cyanoacetate react in the presence of a catalyst to form the ethyl 3-amino-1-aryl-1*H*-benzo[*f*]chromene-2-carboxylates; the compounds possess a primary amine group. The crystal structures of several 1-aryl derivatives have been reported (Klokol *et al.*, 1987; Shi *et al.*, 2003*a*; Shi *et al.*, 2003*b*; Wang *et al.*, 2003; Zhuang *et al.*, 2003*a*; Zhuang *et al.*, 2003*b*). Interestingly, the crystal structure of the unsubstituted 1-phenyl compound was not known, and its structure is reported here. The title compound (Scheme I, Fig. 1) exhibits a pyranyl ring in a flattened boat conformation. The molecule also features an intramolecular *N*–*H*···O_carbonyl_ bond. Adjacent molecules are linked by an intermolecular N—H···O_carbonyl_ hydrogen bond to furnish a zigzag chain that runs along the *c*-axis.

### Experimental

2-Naphthol (1.4 g, 10 mmol), benzaldehyde (1.1 g, 10 mmol), ethyl 2-cyanoacetate (1.0 g, 10 mmol) and piperidine (1 ml) were dissolved in ethanol (30 ml). The solution was heated for 5 h. The solvent was removed under reduced pressure and and the residue recrystallized from dichloromethane/methanol (1:1/v:v) to give yellow crystals in 80% yield; m.p. 436–437 K.

### Refinement

In the absence of significant anomalous scattering Friedel pairs were merged prior to refinement and the absolute configuration of the molecule was not refined. Carbon-bound H atoms were placed in calculated positions [C—H 0.95–1.00 Å and *U*_iso_(H) 1.2–1.5*U*_eq_(C)], and were included in the refinement in the riding-model approximation. The amino H-atoms were located in a difference Fourier map, and were refined with an N–H distance restraint of 0.88 (1) Å; their isotropic temperature factures were refined.

### Crystal data

**Table:**

C_22_H_19_NO_3_	*F*(000) = 728
*M**_r_* = 345.38	*D*_x_ = 1.299 Mg m^−^^3^
Monoclinic, *C**c*	Mo *K*α radiation, λ = 0.71073 Å
Hall symbol: C -2yc	Cell parameters from 8058 reflections
*a* = 13.7835 (5) Å	θ = 3.0–27.5°
*b* = 14.6460 (4) Å	µ = 0.09 mm^−^^1^
*c* = 8.8713 (2) Å	*T* = 153 K
β = 99.551 (1)°	Block, yellow
*V* = 1766.05 (9) Å^3^	0.36 × 0.25 × 0.14 mm
*Z* = 4	

### Data collection

**Table:**

Rigaku R-AXIS RAPID diffractometer	2029 independent reflections
Radiation source: fine-focus sealed tube	1958 reflections with *I* > 2σ(*I*)
graphite	*R*_int_ = 0.016
Detector resolution: 10.000 pixels mm^-1^	θ_max_ = 27.5°, θ_min_ = 3.0°
ω scans	*h* = −17→17
Absorption correction: multi-scan (*ABSCOR*; Higashi, 1995)	*k* = −18→18
*T*_min_ = 0.864, *T*_max_ = 0.988	*l* = −10→11
8563 measured reflections	

### Refinement

**Table:**

Refinement on *F*^2^	Primary atom site location: structure-invariant direct methods
Least-squares matrix: full	Secondary atom site location: difference Fourier map
*R*[*F*^2^ > 2σ(*F*^2^)] = 0.030	Hydrogen site location: inferred from neighbouring sites
*wR*(*F*^2^) = 0.078	H atoms treated by a mixture of independent and constrained refinement
*S* = 1.09	*w* = 1/[σ^2^(*F*_o_^2^) + (0.0522*P*)^2^ + 0.3553*P*] where *P* = (*F*_o_^2^ + 2*F*_c_^2^)/3
2029 reflections	(Δ/σ)_max_ = 0.001
244 parameters	Δρ_max_ = 0.19 e Å^−^^3^
4 restraints	Δρ_min_ = −0.27 e Å^−^^3^

### Fractional atomic coordinates and isotropic or equivalent isotropic displacement parameters (Å^2^)

**Table:**

	*x*	*y*	*z*	*U*_iso_*/*U*_eq_	
O1	0.50000 (10)	0.89966 (8)	0.50000 (13)	0.0251 (3)	
O2	0.57192 (9)	0.73346 (8)	0.07916 (12)	0.0221 (3)	
O3	0.57203 (10)	0.88667 (8)	0.05998 (13)	0.0243 (3)	
N1	0.54563 (13)	0.98073 (10)	0.31277 (17)	0.0289 (3)	
H11	0.5480 (18)	1.0271 (12)	0.376 (2)	0.038 (7)*	
H12	0.5641 (18)	0.9858 (18)	0.2234 (17)	0.040 (7)*	
C1	0.53026 (11)	0.72524 (11)	0.36834 (17)	0.0176 (3)	
H1	0.4968	0.6810	0.2908	0.021*	
C2	0.63064 (12)	0.68559 (10)	0.43558 (18)	0.0199 (3)	
C3	0.66322 (13)	0.60409 (11)	0.3813 (2)	0.0254 (4)	
H3	0.6228	0.5728	0.3004	0.031*	
C4	0.75447 (14)	0.56763 (13)	0.4441 (3)	0.0335 (4)	
H4	0.7759	0.5118	0.4060	0.040*	
C5	0.81378 (14)	0.61264 (14)	0.5619 (3)	0.0364 (5)	
H5	0.8756	0.5874	0.6056	0.044*	
C6	0.78287 (14)	0.69460 (15)	0.6159 (2)	0.0357 (5)	
H6	0.8239	0.7260	0.6959	0.043*	
C7	0.69180 (13)	0.73093 (13)	0.5532 (2)	0.0276 (4)	
H7	0.6711	0.7871	0.5908	0.033*	
C8	0.46549 (12)	0.73777 (11)	0.48933 (17)	0.0191 (3)	
C9	0.41664 (12)	0.66266 (12)	0.54835 (17)	0.0204 (3)	
C10	0.43046 (13)	0.57101 (12)	0.50594 (18)	0.0247 (3)	
H10	0.4732	0.5580	0.4349	0.030*	
C11	0.38294 (15)	0.50035 (13)	0.5660 (2)	0.0305 (4)	
H11A	0.3941	0.4392	0.5378	0.037*	
C12	0.31800 (14)	0.51858 (14)	0.6691 (2)	0.0322 (4)	
H12A	0.2840	0.4698	0.7079	0.039*	
C13	0.30372 (14)	0.60584 (14)	0.7134 (2)	0.0297 (4)	
H13	0.2601	0.6172	0.7837	0.036*	
C14	0.35287 (12)	0.68005 (13)	0.65636 (19)	0.0240 (4)	
C15	0.34044 (13)	0.77096 (13)	0.7054 (2)	0.0278 (4)	
H15	0.2974	0.7827	0.7764	0.033*	
C16	0.38932 (14)	0.84127 (13)	0.65223 (19)	0.0262 (4)	
H16	0.3816	0.9018	0.6867	0.031*	
C17	0.45205 (12)	0.82298 (12)	0.54462 (18)	0.0217 (3)	
C18	0.53012 (12)	0.89655 (11)	0.36102 (17)	0.0213 (3)	
C19	0.53928 (12)	0.81562 (10)	0.28807 (18)	0.0186 (3)	
C20	0.56246 (12)	0.81763 (10)	0.13523 (18)	0.0185 (3)	
C21	0.59893 (13)	0.72831 (11)	−0.07209 (18)	0.0239 (3)	
H21A	0.6644	0.7561	−0.0717	0.029*	
H21B	0.5502	0.7611	−0.1475	0.029*	
C22	0.60081 (17)	0.62865 (13)	−0.1116 (2)	0.0336 (4)	
H22A	0.5345	0.6031	−0.1183	0.050*	
H22B	0.6459	0.5964	−0.0321	0.050*	
H22C	0.6232	0.6214	−0.2101	0.050*	

### Atomic displacement parameters (Å^2^)

**Table:**

	*U*^11^	*U*^22^	*U*^33^	*U*^12^	*U*^13^	*U*^23^
O1	0.0364 (7)	0.0209 (6)	0.0193 (5)	−0.0010 (5)	0.0086 (5)	−0.0031 (4)
O2	0.0328 (6)	0.0182 (5)	0.0168 (5)	0.0025 (5)	0.0084 (4)	0.0014 (4)
O3	0.0334 (7)	0.0200 (6)	0.0199 (5)	0.0007 (5)	0.0059 (5)	0.0027 (4)
N1	0.0473 (9)	0.0188 (7)	0.0216 (7)	−0.0023 (6)	0.0088 (6)	−0.0033 (6)
C1	0.0189 (7)	0.0185 (7)	0.0156 (6)	0.0004 (5)	0.0036 (5)	0.0004 (5)
C2	0.0194 (7)	0.0223 (7)	0.0190 (7)	−0.0004 (6)	0.0061 (5)	0.0069 (6)
C3	0.0223 (8)	0.0188 (7)	0.0357 (10)	−0.0010 (6)	0.0060 (7)	0.0053 (6)
C4	0.0243 (8)	0.0223 (8)	0.0553 (12)	0.0029 (7)	0.0108 (8)	0.0109 (8)
C5	0.0202 (8)	0.0387 (11)	0.0489 (12)	0.0022 (7)	0.0012 (8)	0.0183 (9)
C6	0.0245 (9)	0.0507 (12)	0.0298 (9)	−0.0019 (8)	−0.0014 (7)	0.0060 (8)
C7	0.0236 (8)	0.0359 (9)	0.0230 (8)	−0.0006 (7)	0.0026 (6)	0.0006 (7)
C8	0.0187 (7)	0.0215 (7)	0.0168 (7)	0.0006 (6)	0.0024 (6)	0.0015 (6)
C9	0.0187 (7)	0.0267 (8)	0.0155 (6)	−0.0001 (6)	0.0017 (5)	0.0015 (6)
C10	0.0279 (8)	0.0250 (8)	0.0221 (8)	−0.0044 (6)	0.0067 (6)	0.0003 (6)
C11	0.0365 (10)	0.0277 (8)	0.0272 (9)	−0.0071 (8)	0.0053 (7)	0.0014 (7)
C12	0.0309 (9)	0.0365 (10)	0.0296 (9)	−0.0103 (7)	0.0059 (7)	0.0065 (8)
C13	0.0225 (8)	0.0440 (10)	0.0239 (8)	−0.0020 (7)	0.0078 (6)	0.0057 (7)
C14	0.0190 (7)	0.0320 (9)	0.0208 (8)	0.0011 (6)	0.0030 (6)	0.0024 (6)
C15	0.0241 (8)	0.0376 (10)	0.0233 (8)	0.0063 (7)	0.0085 (6)	−0.0007 (7)
C16	0.0277 (8)	0.0276 (9)	0.0240 (8)	0.0056 (7)	0.0063 (7)	−0.0033 (7)
C17	0.0228 (8)	0.0251 (8)	0.0173 (7)	0.0017 (6)	0.0031 (6)	0.0007 (6)
C18	0.0254 (8)	0.0226 (8)	0.0153 (7)	0.0002 (6)	0.0020 (6)	0.0005 (6)
C19	0.0206 (7)	0.0177 (7)	0.0169 (7)	0.0001 (5)	0.0016 (6)	0.0018 (5)
C20	0.0199 (7)	0.0173 (7)	0.0180 (7)	0.0018 (5)	0.0021 (5)	0.0012 (5)
C21	0.0330 (9)	0.0233 (8)	0.0173 (8)	0.0011 (7)	0.0092 (6)	−0.0003 (6)
C22	0.0519 (11)	0.0238 (8)	0.0280 (9)	0.0062 (8)	0.0155 (8)	−0.0017 (7)

### Geometric parameters (Å, °)

**Table:**

O1—C18	1.3654 (19)	C8—C9	1.433 (2)
O1—C17	1.393 (2)	C9—C10	1.415 (2)
O2—C20	1.3437 (19)	C9—C14	1.427 (2)
O2—C21	1.4527 (18)	C10—C11	1.378 (2)
O3—C20	1.231 (2)	C10—H10	0.9500
N1—C18	1.334 (2)	C11—C12	1.407 (3)
N1—H11	0.876 (10)	C11—H11A	0.9500
N1—H12	0.875 (10)	C12—C13	1.361 (3)
C1—C8	1.517 (2)	C12—H12A	0.9500
C1—C19	1.518 (2)	C13—C14	1.418 (3)
C1—C2	1.528 (2)	C13—H13	0.9500
C1—H1	1.0000	C14—C15	1.420 (3)
C2—C3	1.389 (2)	C15—C16	1.357 (3)
C2—C7	1.396 (2)	C15—H15	0.9500
C3—C4	1.395 (2)	C16—C17	1.416 (2)
C3—H3	0.9500	C16—H16	0.9500
C4—C5	1.382 (3)	C18—C19	1.366 (2)
C4—H4	0.9500	C19—C20	1.444 (2)
C5—C6	1.386 (3)	C21—C22	1.502 (2)
C5—H5	0.9500	C21—H21A	0.9900
C6—C7	1.392 (3)	C21—H21B	0.9900
C6—H6	0.9500	C22—H22A	0.9800
C7—H7	0.9500	C22—H22B	0.9800
C8—C17	1.365 (2)	C22—H22C	0.9800
			
C18—O1—C17	117.34 (13)	C12—C11—H11A	119.9
C20—O2—C21	116.42 (12)	C13—C12—C11	120.25 (17)
C18—N1—H11	120.2 (17)	C13—C12—H12A	119.9
C18—N1—H12	117.1 (17)	C11—C12—H12A	119.9
H11—N1—H12	122 (2)	C12—C13—C14	121.08 (17)
C8—C1—C19	109.27 (13)	C12—C13—H13	119.5
C8—C1—C2	111.78 (13)	C14—C13—H13	119.5
C19—C1—C2	112.04 (12)	C13—C14—C15	121.35 (16)
C8—C1—H1	107.9	C13—C14—C9	119.15 (16)
C19—C1—H1	107.9	C15—C14—C9	119.50 (15)
C2—C1—H1	107.9	C16—C15—C14	120.85 (16)
C3—C2—C7	118.56 (15)	C16—C15—H15	119.6
C3—C2—C1	121.00 (14)	C14—C15—H15	119.6
C7—C2—C1	120.44 (15)	C15—C16—C17	118.99 (16)
C2—C3—C4	120.82 (17)	C15—C16—H16	120.5
C2—C3—H3	119.6	C17—C16—H16	120.5
C4—C3—H3	119.6	C8—C17—O1	122.40 (14)
C5—C4—C3	120.03 (18)	C8—C17—C16	123.36 (16)
C5—C4—H4	120.0	O1—C17—C16	114.24 (14)
C3—C4—H4	120.0	N1—C18—O1	110.36 (14)
C4—C5—C6	119.83 (17)	N1—C18—C19	128.13 (15)
C4—C5—H5	120.1	O1—C18—C19	121.50 (14)
C6—C5—H5	120.1	C18—C19—C20	118.64 (14)
C5—C6—C7	120.10 (18)	C18—C19—C1	120.87 (14)
C5—C6—H6	120.0	C20—C19—C1	120.42 (13)
C7—C6—H6	120.0	O3—C20—O2	121.82 (15)
C6—C7—C2	120.65 (18)	O3—C20—C19	125.89 (14)
C6—C7—H7	119.7	O2—C20—C19	112.28 (13)
C2—C7—H7	119.7	O2—C21—C22	106.43 (13)
C17—C8—C9	118.06 (14)	O2—C21—H21A	110.4
C17—C8—C1	119.73 (14)	C22—C21—H21A	110.4
C9—C8—C1	122.20 (14)	O2—C21—H21B	110.4
C10—C9—C14	118.23 (15)	C22—C21—H21B	110.4
C10—C9—C8	122.59 (15)	H21A—C21—H21B	108.6
C14—C9—C8	119.17 (15)	C21—C22—H22A	109.5
C11—C10—C9	121.04 (16)	C21—C22—H22B	109.5
C11—C10—H10	119.5	H22A—C22—H22B	109.5
C9—C10—H10	119.5	C21—C22—H22C	109.5
C10—C11—C12	120.21 (18)	H22A—C22—H22C	109.5
C10—C11—H11A	119.9	H22B—C22—H22C	109.5
			
C8—C1—C2—C3	121.86 (16)	C8—C9—C14—C15	−1.2 (2)
C19—C1—C2—C3	−115.10 (16)	C13—C14—C15—C16	178.73 (17)
C8—C1—C2—C7	−58.27 (19)	C9—C14—C15—C16	−0.8 (3)
C19—C1—C2—C7	64.76 (19)	C14—C15—C16—C17	1.0 (3)
C7—C2—C3—C4	0.8 (2)	C9—C8—C17—O1	176.85 (14)
C1—C2—C3—C4	−179.31 (16)	C1—C8—C17—O1	−3.5 (2)
C2—C3—C4—C5	0.0 (3)	C9—C8—C17—C16	−2.7 (2)
C3—C4—C5—C6	−0.8 (3)	C1—C8—C17—C16	176.99 (16)
C4—C5—C6—C7	0.8 (3)	C18—O1—C17—C8	24.8 (2)
C5—C6—C7—C2	0.0 (3)	C18—O1—C17—C16	−155.62 (15)
C3—C2—C7—C6	−0.8 (3)	C15—C16—C17—C8	0.8 (3)
C1—C2—C7—C6	179.31 (16)	C15—C16—C17—O1	−178.81 (15)
C19—C1—C8—C17	−20.86 (19)	C17—O1—C18—N1	160.53 (15)
C2—C1—C8—C17	103.73 (16)	C17—O1—C18—C19	−18.3 (2)
C19—C1—C8—C9	158.81 (13)	N1—C18—C19—C20	−4.4 (3)
C2—C1—C8—C9	−76.60 (18)	O1—C18—C19—C20	174.24 (15)
C17—C8—C9—C10	−175.95 (15)	N1—C18—C19—C1	172.70 (16)
C1—C8—C9—C10	4.4 (2)	O1—C18—C19—C1	−8.7 (2)
C17—C8—C9—C14	2.8 (2)	C8—C1—C19—C18	27.1 (2)
C1—C8—C9—C14	−176.84 (15)	C2—C1—C19—C18	−97.31 (17)
C14—C9—C10—C11	0.6 (2)	C8—C1—C19—C20	−155.85 (14)
C8—C9—C10—C11	179.41 (15)	C2—C1—C19—C20	79.71 (17)
C9—C10—C11—C12	1.2 (3)	C21—O2—C20—O3	3.0 (2)
C10—C11—C12—C13	−1.8 (3)	C21—O2—C20—C19	−177.75 (13)
C11—C12—C13—C14	0.5 (3)	C18—C19—C20—O3	−2.5 (2)
C12—C13—C14—C15	−178.19 (18)	C1—C19—C20—O3	−179.59 (16)
C12—C13—C14—C9	1.3 (3)	C18—C19—C20—O2	178.28 (15)
C10—C9—C14—C13	−1.9 (2)	C1—C19—C20—O2	1.20 (19)
C8—C9—C14—C13	179.31 (15)	C20—O2—C21—C22	−177.74 (14)
C10—C9—C14—C15	177.66 (15)		

### Hydrogen-bond geometry (Å, °)

**Table:**

*D*—H···*A*	*D*—H	H···*A*	*D*···*A*	*D*—H···*A*
N1—H11···O3^i^	0.88 (1)	2.05 (1)	2.907 (2)	166 (2)
N1—H12···O3	0.88 (1)	2.07 (2)	2.707 (2)	129 (2)

Symmetry codes: (i) *x*, −*y*+2, *z*+1/2.
